# Comparison of off-pump and on-pump coronary endarterectomy for patients with diffusely diseased coronary arteries: early and midterm outcome

**DOI:** 10.1186/s13019-014-0186-5

**Published:** 2014-12-04

**Authors:** Zhibing Qiu, Xin Chen, YingShou Jiang, LiMing Wang, Ming Xu, Fuhua Huang, Hongwei Shi, Cui Zhang

**Affiliations:** Department of Cardiothoracic Surgery, Nanjing First Hospital, Nanjing Medical University, Nanjing Heart Institute, 68 Changle Rd, Nanjing, 210006 China

**Keywords:** Coronary endarterectomy, Off-pump, On-pump, Diffused coronary disease, Left internal mammary artery

## Abstract

**Background:**

Coronary endarterectomy (CE) is an alternative for the diffusely diseased left anterior descending (LAD), but its mid and long term results are largely questionable. This study is to compare the early to mid-term results between off-pump and on-pump coronary endarterectomy with coronary artery bypass grafting.

**Methods:**

212 consecutive patients underwent CE and bypass grafting for diffusely diseased LAD. Ninety-two patients undergoing CE with off-pump (group off-pump) were compared with 120 patients undergoing CE with on-pump (group on-pump). The main preference for selection to an off-pump CE surgery were the preoperative high risk factors, especially previous cerebrovascular accident、chronic obstructive pulmonary disease (COPD)、calcified ascending aorta and right coronary artery (RCA) critical stenosis >90%.

**Results:**

There were three deaths in this group with total operative mortality of 1.4%. The perioperative mortality of group off-pump (1.1%) was similar with that of group on-pump (1.7%). The postoperative myocardial infarctions rate was 2.8%. There was no significant difference as for the morbidity between the group off-pump and group on-pump. Among survivors, the patency rate of the LIMA–LAD anastomosis was 89.4%. There was no difference as for the grafts patency rate between the two groups. Kaplan–Meier survival revealed no significant difference between the two groups. Kaplan-Meier freedom from cardiac events requiring hospital re-admission and angina recurrence were similar in both groups.

**Conclusions:**

On-pump or off-pump CE is a good technique with the same early and mid-term outcomes. In the series of off-pump CE, we have shown that the effect of OPCABG with CE appears to be durable, and mid-term clinical outcomes are encouraging. Despite the higher risk profile, hospital mortality and major complications in our study are comparable to those for CCE.

**Electronic supplementary material:**

The online version of this article (doi:10.1186/s13019-014-0186-5) contains supplementary material, which is available to authorized users.

## Background

Coronary endarterectomy (CE), first described by Bailey et al. in 1957, has been shown to benefit patients with advanced coronary atheroma by providing complete revascularization [1]. The safety and long-term efficacy of the procedure, although controversial, has been demonstrated in earlier studies [2],[3]. Today, many surgeons are still reluctant to use coronary endarterectomy because of increased mortality and postoperative myocardial infarction (MI) rates in comparison with coronary artery bypass grafting (CABG) alone [4],[5].

Off-pump coronary endarterectomy has also been reported, but the literature is limited [6]. The advent of safe off-pump coronary bypass (OPCAB) with better stabilization of the target vessels has renewed our interest in this procedure in selected patients [7],[8]. On-pump or off-pump endarterectomy helps achieve effective revascularization of vessels that otherwise appear to be inoperable. However, we should consider the various techniques, apparent increase in myocardial infarction rates, vessel rupture due to inadequate traction, long-term patency rates below those of vessels without endarterectomy, and mortality rates, in order to offer optimal revascularization in patients who cannot receive percutaneous angioplasty and to provide a surgical option for patients who would otherwise be inoperable.

Herein, we report our experience with this procedure, in an effort to examine its efficacy and the early to mid-term outcomes in two groups of patients who underwent off-pump and on-pump coronary endarterectomy for patients with diffusely diseased left anterior descending artery(LAD).

## Methods

### Patients

Between January 1999 and December 2013, of the 4328 patients who underwent CABG, 212 patients underwent myocardial revascularization with CE at the Nanjing first hospital to achieve a complete revascularization. The Ethics committee approved relating screening, treatment, and data collection of these patients, all cases signed written informed consent form. All works were undertaken following the provisions of the Declaration of Helsinki.

There were 180 (84.9%) men and 32 (15.1%) women, with a mean age of 68.7 ± 7.5 years (range, 45–78 years). The risk factors include diabetes in 176(83.0%), hypertension in 134(63.2%), hyperlipidemia in 107(50.5%), peripheral artery disease in 39(18.4%), and history of smoking in 95(44.8%) patients. Previous myocardial infarction had been experienced in 137 (64.6%) patients. Unstable angina was present in 155 (73.1%) patients. The left ventricular ejection fraction (LVEF) was 0.50 ± 0.13 (0.23-0.61). Impairment of the ventricular function (LVEF<35%) was present in 19 (8.9%) patients. 33 (15.6%) patients had undergone a previous percutaneous transluminal coronary angioplasty (PTCA) (Table 1).

**Table 1:** **Preoperative patient characteristics**

Variable	Group on-pump (n = 120)	Group off-pump (n = 92)	***P***value
Clinical demographics			
Age (y)	65 ± 8	63 ± 9	NS
Female sex	20 (16.6%)	12 (13.0%)	NS
Coronary risk factors			
Hypertension	75 (62.5%)	59 (64.1%)	NS
Diabetes	100 (83.3%)	76 (82.6%)	NS
Hyperlipidemia	57 (47.5%)	50 (54.3%)	<0.05
Smoking	55 (45.8%)	41 (44.6%)	NS
Peripheral vascular disease	22 (18.3%)	17 (18.5%)	NS
Previous renal impairment	6 (5.0%)	4 (4.3%)	NS
Cerebrovascular accident	2 (1.7%)	8 (8.7%)	<0.001
COPD	5 (4.2%)	22 (23.9%)	<0.001
Calcified ascending aorta	2 (1.7%)	45 (48.9%)	<0.001
Cardiac profile			
Previous myocardial infarction	77 (64.2%)	59 (64.1%)	NS
Unstable angina	87 (72.5%)	68 (73.9%)	NS
previous PTCA	19 (15.8%)	15 (16.3%)	NS
Mean ejection fraction	0.50 ± 0.12	0.49 ± 0.14	NS
Poor ejection fraction (<0.35)	13 (10.8%)	9 (9.8%)	NS
Left main disease	23 (19.2%)	17 (18.5%)	NS
RCA Critical stenosis >90%	68 (56.7%)	89 (96.7%)	<0.001
Number of diseased vessels	3.8 ± 0.8	3.9 ± 0.9	NS

NS = not significant. PTCA = percutaneous transluminal coronary angioplasty.

### Surgical technique for coronary endarterectomy

This study includes 212 patients with diffusely diseased LAD artery requiring operation for myocardial revascularization. LAD endarterectomy and bypass grafting were done with cardiopulmonary bypass (CPB) (on-pump) in 120 patients and without CPB in the beating heart (Off-pump) in 92 cases. Complete revascularization was aimed for in all patients. All operations were carried out through a full sternotomy. The left internal mammary artery (LIMA) was prepared in all cases. All the patients were operated on by a single surgeon (Dr. Xin Chen). The anaesthesia, perfusion, surgical and postoperative care team also remained the same for each case.

The decision to perform endarterectomy was based on the preoperative angiograms and intra-operative findings. The CE was scheduled before operation, but the final decision of applying which technique was made during the operation according to the surgeon’s preference. The main reasons for choosing an off-pump procedure were the preoperative high risk factors shown Table 1, especially previous cerebrovascular accident、chronic obstructive pulmonary disease (COPD)、calcified ascending aorta and right coronary artery(RCA) critical stenosis >90%.

An open extended arteriotomy with patch angioplasty was used in all procedures. As much as possible plaque was removed. If residual plaque was thought to remain in the distal vessel, the arteriotomy was enlarged as much as necessary to allow complete removal of the plaque, especially on the LAD. The mean lengths of arteriotomies were 14.3 ± 2.6 mm (range 10–22 mm). None of the cases required a second arteriotomy distal to the first. When a long arteriotomy and a LIMA graft were used, a vein patch was typically applied to the endarterectomized bed, and the LIMA was then applied to the hood of the vein patch.

In group off-pump, all procedures were performed through a median sternotomy. After the conduits were harvested, heparin was administered to maintain an activated clotting time greater than 250 seconds. Three deep left pericardial sutures were used for cardiac exposure, and a suction device (Octopus Evolution, Medtronic, Minneapolis, MN) was used for stabilization of the coronary arteries. A shunt (Chase Medical, Richardson, TX) was inserted in the coronary artery during all anastomoses to avoid ischemic damage and perioperative rhythm disturbances. A blower/mister was systematically used to obtain a bloodless operative field and perfect visualization of the coronary artery.

In group on-pump, All procedures were performed with extracorporeal circulationand moderate hypothermia (31°C); myocardial protection wasobtained by means of intermittent antegrade crystalloid cardioplegia and topical cooling with coldsaline (4°C).

In this study, the grafts used include LIMA, radial artery, and saphenous veins. Intra-operative Medi-Stim Butterfly flowmeter (Medi-Stim AS, Oslo, Norway) were used to check the flow of the grafts before chest closure. Anticoagulation therapy was performed with a combination of aspirin and clopidogrel. Clopidogrel is continued for 6 months postoperatively and Aspirin lifelong. Patients in atrial fibrillation or those with mechanical valves are still anticoagulated with Warfarin and aspirin.

### Data collection and follow-Up

Demographic variables, preoperative symptom status, risk factors, operative details and postoperative events indicating morbidity and mortality were recorded. Preoperative risk factors for coronary artery disease were studied along with postoperative out come for safety, morbidity and mortality in these patients. Postoperative myocardial infarction (MI) was established by new Q waves or conduction blocks on an electrocardiogram with creatine kinase MB (CK-MB) more than 100 mg · d/L and/or CK-MB/total CK ratio > 10% in the postoperative 24th hour. A new regional wall motion abnormality on echo was also taken as evidence of postoperative MI. Complete follow-up was obtained for survival. Patients followed up at regular intervals in the outpatient clinic and the latest follow up was obtained by telephone interview at the start of the study with regards to their current symptom status, need for nitrates, hospital readmissions, and re-interventions, and any surgery-related complications.

### Graft patency analysis

The quality of the anastomosis was graded according to the classification of Fitzgibbon and colleagues [9]. Briefly, grade A stands for excellent graft patency, grade B for graft stenosis of greater than 50%, and grade O for occlusion. Follow up of the grafts were done in all surviving patients by coronary angiograph in 21 and 64 slices CT scan in 48 cases after the operation.

### Statistical analysis

Statistical analysis was performed by using SPSS 15.0 statistical software (SPSS Inc, Chicago, Ill). Continuous variables were expressed as mean ± SD, and categoric variables were expressed as percentages. Basic methods of univariate analysis included the impaired 2-tailed *t* test or Fisher exact test when appropriate. Values of p less than 0.05 were considered significant. Actuarial curves were constructed to describe mortality and the incidence of valve-related complications using the Kaplan-Meier technique. Survival data were analyzed by using the standard Kaplan-Mayer actuarial technique for estimation of survival probabilities.

## Results

### Patient demographics

Preoperative data for both groups are summarized in Table 1. Preoperative risk factors for coronary artery disease were observed in both groups. 48.9% patients were calcified ascending aorta in group off-pump and1.7% were in group on-pump (P<0.001). RCA critical stenosis >90% was present in 96.7% of patients in group off-pump and 56.7% patients in group on-pump (P<0.001). Hyperlipidemia, COPD and previous cerebrovascular accident was present in 54.3%, 23.9%, 8.7% in group off-pump and 47.5%, 4.2% and 1.7% respectively in group on-pump (P<0.001) (Table 1).

### Intraoperative data

The mean number of distal anastomosis is 3.9 ± 0.8. Twenty-three patients had coronary endarterectomy in other coronary territory other than LAD (15 in right coronary and 8 in circumflex artery territory) at the same time. The mean lengths of the arteriotomy were similar in both groups. The LIMA was all used to reconstruct the endarterectomized LAD vessel in two groups, and the other grafts to anastomosis were similar between the two groups (P>0.05; Table 2). The other simultaneous associated procedures include left ventricle (LV) reconstruction for LV aneurysm in 6, and mitral repair or replacement in 13, carotid endarterectomy in 3 patients. 200 out of 212 grafts (94.3%) to the LAD after endarterectomy showed a satisfactory grafts flow intra-operatively (mean flow 30 ± 12 ml/min, PI 2.6 ± 0.8). IABP were needed in 12 patients to stabilize hemodynamic.

**Table 2:** **Operative characteristics**

Variable	Group on-pump (n = 120)	Group off-pump (n = 92)	***P***Value
Length of the ateriotomy	14.5 ± 2.5	14.1 ± 2.8	NS
Operative time (min)	253 ± 42	260 ± 48	NS
The number of distal anastomoses	4.0 ± 0.9	3.8 ± 0.7	NS
Associatied procedure	32 (26.6%)	26 (28.3%)	NS
Intra-aortic balloon pump	9 (7.5%)	3 (3.3%)	0.015
Conduit to anastomosis vessel			
Left internal mammary artery	120 (100%)	92 (100%)	NS
Radial artery	23 (19.1%)	19 (20.7%)	NS
Saphenous vein	113 (94.2%)	88 (95.7%)	NS

NS = not significant.

Operation time, the number of distal anastomoses and associated procedure were similar between the group off-pump and group on-pump. Intra-aortic balloon pump in group off-pump was smaller than that in group on-pump (P<0.05; Table 2).

### Perioperative morbidity and mortality

There were three deaths in this group because of low cardiac output syndrome and renal failure respectively with total operative mortality of 1.4%. The perioperative mortality rate of group off-pump (1.1%) was similar with that of group on-pump (1.7%) (P>0.05; Table 3). Six patients (2.8%) had postoperative myocardial infarctions—in three cases of group off-pump (3.3%) and in three cases of group on-pump (2.5%)—but none had hemodynamic changes except one. The perioperative ventricular arrhythmia rate of group off-pump (8.1%) tended to be lower than that of group on-pump (9.5%) (P<0.05). Two patients were re-explored because of bleeding. The fatal postoperative complication rates were similar, and there were no statistically significant differences between the groups (P>0.05).

**Table 3:** **Perioperative mortality and morbidity**

Variable	Group on-pump (n = 120)	Group off-pump (n = 92)	***P***Value
Death (30-day)	2 (1.7%)	1 (1.1%)	NS
Perioperative myocardial infarction	3 (2.5%)	3 (3.3%)	NS
Ventricular arrhythmia	8 (6.7%)	11 (12.0%)	<0.05
Re-exploration for bleeding	1 (0.8%)	1 (1.1%)	NS
Cerebral vascular accident	1 (0.8%)	1 (1.1%)	NS
Acute renal failure	6 (5.0%)	5 (5.4%)	NS
Intensive care unit stay (days)	2.5 ± 0.5	2.6 ± 0.7	NS
Infection	3 (2.5%)	2 (2.2%)	NS

NS = not significant.

### Survival analysis

**Figure 1:**
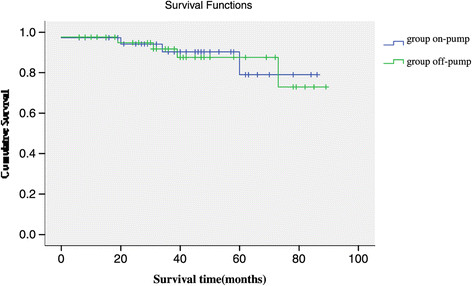
**Mid-term survival with group on-pump versus group off-pump after coronary endarterectomy.**

209 patients discharged uneventfully with mean hospital stay 9.7 days postoperatively. There were 7 late deaths overall: 3 patients (3.3%) in group off-pump, and 4 patients (3.4%) in group on-pump. Kaplan–Meier survival estimates at 1, 3, and 5 years were 0.942, 0.902, and 0.790 in group off-pump, and 0.948, 0.875, and 0.729 in group on-pump (Figure 1). However, comparison of survival curves between the two groups revealed no significant difference.

### Mid-term outcomes and graft patency analysis

**Figure 2:**
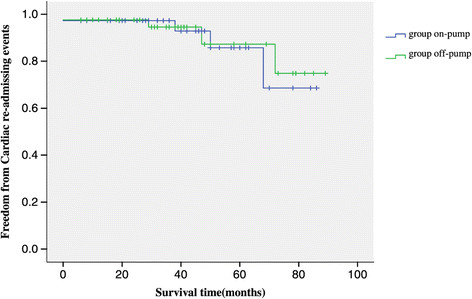
**Kaplan-Meier Freedom from cardiac events requiring hospital re-admission.**

**Figure 3:**
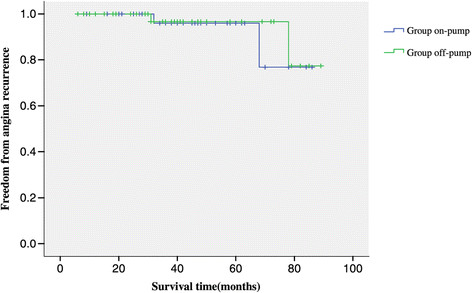
**Kaplan-Meier Freedom from angina recurrence.**

**Figure 4:**
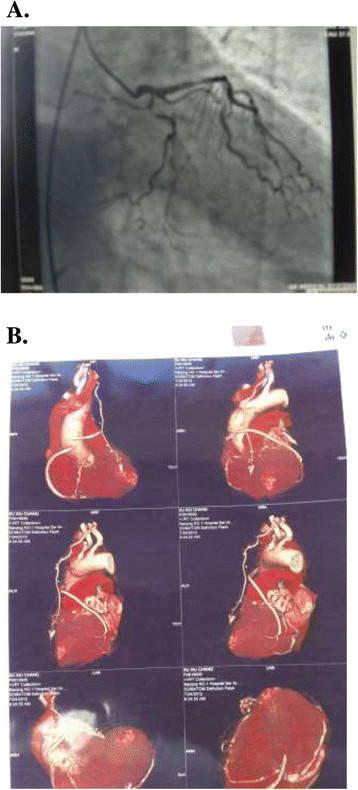
**Preoperative and Postoperative follow-up angiogram study. (A)** Preoperative angiogram demonstrates a diffusely diseased left anterior descending coronary artery (LAD). **(B)** Postoperative follow-up computed tomography (CT) angiogram showing patency of the left internal mammary artery to the LAD grafts.

209 patients had a mean follow up 41.5 ± 22.0 (range, 6 to 89) months. When patients were compared, Actuarial freedom from cardiac events requiring hospital re-admission was 92.2% (Figure 2), and freedom from angina recurrence was 94.8% (Figure 3). Kaplan-Meier freedom from cardiac events requiring hospital re-admission (Figure 2) and angina recurrence (Figure 3) were similar in both groups. At mid-term follow-up, all patients underwent postoperative angiographic study, and118 grafts (89.4%) out of 132 patients with satisfactory grafts flow were patent (Figure 4). Information regarding coronary angiograph or 64 slices CT scan was obtained from survivors of group off-pump (mean, 41.8 ± 21.4 months; range, 6 to 86 months) and of group on-pump (mean, 41.2 ± 22.7 months; range, 6 to 89 months). There is no difference as for the grafts early and midterm patency rate between the group off-pump and group on-pump (Table 4).

**Table 4:** **Follow-up graft patency analysis**

Variable	Group on-pump	Group off-pump	***P***Value
Early patency rate	94.5% (86/91)	94.9% (112/118)	NS
Grade A	91.8% (79)	92.9% (104)	NS
Grade B	8.1% (7)	7.1% (8)	NS
Midterm patency rate	91.7% (55/60)	90.3% (65/72)	NS
Grade A	90.9% (50)	89.2% (58)	NS
Grade B	9.1% (5)	10.8% (6)	NS

NS = not significant.

## Discussion

The completeness of revascularization has been shown to correlate with improved early and late patient outcomes after CABG [6],[9]. Proper and complete revascularisation of the coronaries especially the LAD is the most important determinant of the patient prognosis [10]-[12].

Currently, in patients with end-stage coronary atherosclerosis, the options available include performing CABG to small distal vessels, not performing any bypass to the diffusely diseased coronary territory or using CE as an adjunctive tool to CABG to achieve a complete revascularization [7],[13],[14].

However, CE is reported as a time-consuming and challenging procedure, and its beneficial effect has been questioned because an increased risk of perioperative mortality and morbidity often accompanies this technique, especially when performed on the LAD [4],[15]. In our institution CE was carried out on a small group of highly selected patients when distal anastomosis to an adequately sized vessel was not possible.

Concerns about technical difficulties and adequacy of endarterectomy expose patients to the risks of CPB. Coronary endarterectomy without associated CABG was introduced to treat coronary artery disease and was performed initially without cardiopulmonary bypass [16]. Due to increasing age, preoperative renal, pulmonary, and neurological problems are encountered more often. Off-pump CABG has many advantages in high-risk patients. With increasing use of off-pump coronary bypass, isolated groups have reported CE in this subgroup with good initial results [17],[18]. In this study, coronary artery endarterectomy and bypass grafting for diffusely diseased LAD with or without cardiopulmonary bypass is technically feasible, and can be performed safely with increased completeness of myocardial revascularization but without increasing morbidity and mortality. Owing to the increasing number of patients with diffuse coronary artery disease being referred for OPCABG, there is a need to reassess the early and medium-term postoperative outcomes in patients undergoing primary OPCABG with CE in modern cardiac surgery. The use of OPCABG has found a wide acceptance especially in high-risk patients. It is known to reduce the incidence of renal, neurologic, and inflammatory adverse effects of cardiopulmonary bypass. Naseri et al. reported a reduction in postoperative hospital stay and mortality in patients where coronary endarterectomy was performed off pump compared with the coronary endarterectomy performed using cardiopulmonary bypass, although there was an increased incidence of myocardial infarction in the off-pump patients [16]. With recent advances in the CE technique, the perioperative myocardial infarction rates have improved and now range from 1.5% to 8% [2],[5],[19]. Thus, the early myocardial infarction rates and postoperative mortality in our series was acceptable, especially in the case of off pump endarterectomy, which is similar to the early mortality rates reported by other investigators [20],[21]. Cheng DC et al. [22] undertook a meta-analysis of 37 randomized trials (3369 patients) of off-pump coronary artery bypass surgery *versus* conventional coronary artery bypass surgery. This meta-analysis demonstrates that mortality, stroke, myocardial infarction, and renal failure were not reduced in off-pump coronary artery bypass surgery surgery; however, selected short-term and mid-term clinical and resource outcomes were improved compared with conventional coronary artery bypass surgery. In our studys, the early mortality of 1.1% in the off-pump group is similar when compared with on-pump endarterectomy in 120 patients for whom the mortality rate was 1.7%. Our 94.2% 1-year survival, 90.2% 3-year survival, and 79% 5-year survival rates in the off-pump groupare as compared with on-pump CCE. Patients who have a severe diffuse coronary lesion and are a high risk for CPB will benefit from this off-pump technique.

Hence, diffuse disease requiring endarterectomy should not be considered a contraindication to OPCABG. Surgical skills and the suitability criteria of the patients are very important in this regard. The best approach to off-pump endarterecotmy is to sequentially stabilize short segments of the blood vessel. As opposed to a non-beating heart, endarterectomy in this situation requires very deliberate movements in order to avoid inadvertent tearing of tissue [23],[24]. Our technique of CE using on-pump or off-pump can provide the same better match in diameter with the graft, the reconstructed segment, and the native coronary artery, resulting in better flow patterns. Therefore, there was no significant difference of perioperative mortality and morbidity between the two groups. Our technique of on-pump or off-pump grafting reduced the endarterectomized area and presented new coronary artery wall. This might achieve rapid complete endothelial covering and should decrease the risk of intimal proliferation [23]. A simple non-invasive method to determine the presence of a coronary lesion, such as MDCT, is preferred, especially in patients with a high risk [25]. Moreover, there were no obvious differences of graft patency and survival rates between the two groups. In all cases on-pump or off-pump method was used for bypassing the artery. For LAD endarterectomy LIMA bypass was the preferred technique. Off-pump endarterectomy of the LAD is feasible because the severe underlying ischaemia conditions the myocardium to flow interruption for prolonged periods during the endarterectomy [26].

Several limitations of our study need to be recognized. This is a retrospective study of a small non-randomized cohort of patients. A control group of patients with similar disease undergoing conventional surgery would be ideal, but may not be feasible. The absence of clinical ischaemic events, while encouraging, does not prove the patency of the endarterectomy sites. The observational nature of the study prevents us from drawing conclusions other than the feasibility and safety of the technique.

## Conclusions

Coronary endarterectomy along with CABG in selected cases is an optimal way of revascularization in patients with a diffusely diseased coronary artery. In the series of OPCABG with CE, we have shown that the effect of OPCABG with CE appears to be durable, and long-term clinical outcomes are encouraging. Despite the higher risk profile, hospital mortality and major complications in our study are comparable to those for CCE. Coronary endarterectomy should be used whenever possible in selected patients in a programmed manner to promote myocardial revascularization. We conclude that off-pump endarterectomy of the LAD is a viable option for patients with diffuse LAD disease and high risk factors, particularly in centers with considerable OPCAB experience. A long-term follow up is necessary to demonstrate the future performance of these vessels and grafts. In view of the small numbers in our group, this requires to be validated by a multicentre study with larger number of patients.

## Authors’ original submitted files for images

Below are the links to the authors’ original submitted files for images. Authors’ original file for figure 1 Authors’ original file for figure 2 Authors’ original file for figure 3 Authors’ original file for figure 4

## Abbreviations

CE: Coronary endarterectomy
LAD: Left anterior descending
CABG: Coronary artery bypass grafting
OPCAB: Off-pump coronary bypass
MI: Myocardial infarction
LVEF: Left ventricular ejection fraction
CPB: Cardiopulmonary bypass
LIMA: Left internal mammary artery
CK-MB: Creatine kinase MB
CT: Computed tomography

## References

[1] Bailey C, May A, Lemmon W (1957). Survival after coronary endarterectomy in man. JAMA.

[2] Loop FD (1988). Resurgence of coronary endarterectomy. J Am Coll Cardiol.

[3] Christakis GT, Roo V, Frames SE, Chon E, Naylor CD, Goldman BS (1993). Does coronary endarterectomy adversely effect results of bypass surgery?. J Card Surg.

[4] Byrne JG, Karavas AN, Gudbjartson T, Leacche M, Rawn JD, Couper GS, Cohn LH, Aranki SF (2004). Left anterior descending coronary endarterectomy: early and late results in 196 consecutive patients. Ann Thorac Surg.

[5] Minale C, Nikol S, Zander M, Uebis R, Effert S, Messmer BJ (1989). Controversial aspects of coronary endarterectomy. Ann Thorac Surg.

[6] Naseri E, Arsan S (1999). Coronary endarterectomy on beating heart. Ann Thorac Surg.

[7] Angelini GD, Taylor FC, Reeves BC, Ascione R (2002). Early and midterm outcome after off-pump and on-pump surgery in beating heart against cardioplegic arrest studies (BHACAS 1 and 2): a pooled analysis of two randomised controlled trials. Lancet.

[8] Van Dijk D, Nierich AP, Jansen EW, Nathoe HM, Suyker WJ, Diephuis JC, Van Boven WJ, Borst C, Buskens E, Grobbee DE, Robles De Medina EO, De Jaegere PP (2001). Early outcome after off-pump versus on-pump coronary bypass surgery: results from a randomized study. Circulation.

[9] Fitzgibbon GM, Kafka HP, Leach AJ, Keon WJ, Hooper D, Burton JR (1996). Coronary bypass graft fate and patient outcome: angiographic follow-up of 5,065 grafts related to survival and re-operation in 1,388 patients during 25 years. J Am Coll Cardiol.

[10] Marinelli G, Chiappini B, Eusanio MD, Bartolomeo RD, Caldarera I, Marrozzini C, Pierangeli A (2002). Bypass grafting with coronary endarterectomy: immediate and long-term results. J Thorac Cardiovasc Surg.

[11] Atik FA, Dallan LA, de Oliveira SA (2000). Myocardial revascularization with coronary endarterectomy: stratification of risk factors for early mortality. Arq Bras Cardiol.

[12] Gill IS, Beanlands DS, Boyd WD, Finlay S, Keon WJ (1998). Left anterior descending endarterectomy and internal thoracic artery bypass for diffuse coronary disease. Ann Thorac Surg.

[13] Mills NE (1998). C**oronary endarterectomy**. Adv Cardiac Surg.

[14] Ammirati E, Rimoldi OE, Camici PG (2011). Is there evidence supporting coronary revascularization in patients with left ventricular systolic dysfunction?. Circ J.

[15] Sundt TM, Camillo CJ, Mendeloff EN, Barmer HB, Gay WA (1999). Reappraisal of coronary endarterectomy for the treatment of diffuse coronary artery disease. Ann Thorac Surg.

[16] Naseri E, Sevinc M, Erk MK (2003). Comparison of off-pump and conventional coronary endarterectomy. Heart Surg Forum.

[17] Eryilmaz S, BahadirInan M, Akalin H (2003). Coronary endarterectomy with off pump coronary artery bypass surgery. Ann Thorac Surg.

[18] Reyna GC, Garrido DS, Luna ST, Sánchez RA (2003). Coronary endarterectomy and bypass grafting without cardiopulmonary bypass. Rev Esp Cardiol.

[19] Silberman S, Dzigivker I, Merin O, Shapira N, Deeb M, Bitran D (2002). Does coronary endarterectomy increase the risk of coronary bypass. J Card Surg.

[20] Santini F, Casali G, Lusini M, D’Onofrio A, Barbieri E, Rigatelli G, Franco G, Mazzucco A (2002). Mid-term results after extensive vein patch reconstruction and internal mammary grafting of the diffusely diseased left anterior descending coronary artery. Eur J Cardiothorac Surg.

[21] Schmitto JD, Kolat P, Ortmann P, Popov AF, Coskun KO, Friedrich M, Sossalla S, Toischer K, Mokashi SA, Tirilomis T, Baryalei MM, Schoendube FA (2009). Early results of coronary artery bypass grafting with coronary endarterectomy for severe coronary artery disease. J Cardiothorac Surg.

[22] Cheng DC, Bainbridge D, Martin JE, Novick RJ (2005). Does off-pump coronary artery bypass reduce mortality, morbidity and resource utilization when compared with conventional coronary artery bypass? A meta-analysis of randomized trials. Anesthesiology.

[23] Nishi H, Miyamoto S, Takanashi S, Minamimura H, Ishikawa T, Kato Y, Shimizu Y (2005). Optimal method of coronary endarterectomy for diffusely diseased coronary arteries. Ann Thorac Surg.

[24] Vohra HA, Kanwar R, Khan T, Dimitri WR (2006). Early and late outcome after off-pump coronary artery bypass graft surgery with coronary endarterectomy: a single-center 10-year experience. Ann Thorac Surg.

[25] Muto A, Model L, Ziegler K, Eghbalieh SD, Dardik A (2010). Mechanisms of vein graft adaptation to the arterial circulation–insights into the neointimal algorithm and management strategies. Circ J.

[26] Takahashi M, Gohil S, Tong B, Lento P, Filsoufi F, Reddy RC (2013). Early and mid-term results of off-pump endarterectomy of the left anterior descending artery. Interact Cardiovasc Thorac Surg.

