# Supplementary figures and images for: Comparison of off-pump and on-pump coronary endarterectomy for patients with diffusely diseased coronary arteries: early and midterm outcome
Source: J Cardiothorac Surg. 2014 Dec 4;9:186. doi: 10.1186/s13019-014-0186-5 (PMC4266222; doi:10.1186/s13019-014-0186-5)

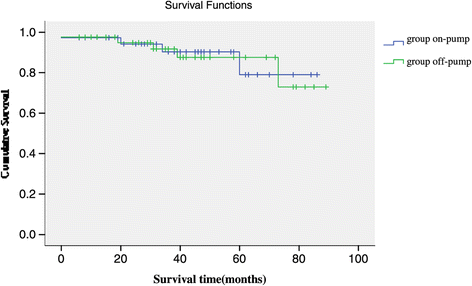

Supplement: Supplementary file 1 — Authors’ original file for figure 1 [file 13019_2014_186_MOESM1_ESM.gif]

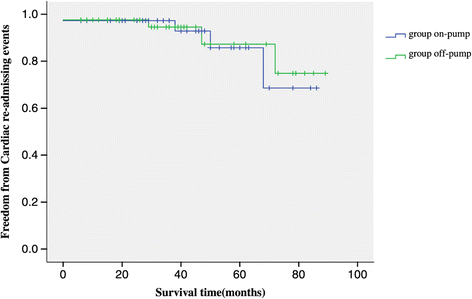

Supplement: Supplementary file 2 — Authors’ original file for figure 2 [file 13019_2014_186_MOESM2_ESM.gif]

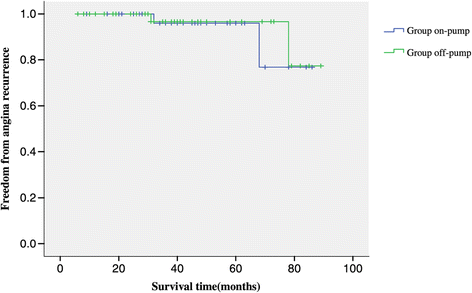

Supplement: Supplementary file 3 — Authors’ original file for figure 3 [file 13019_2014_186_MOESM3_ESM.gif]

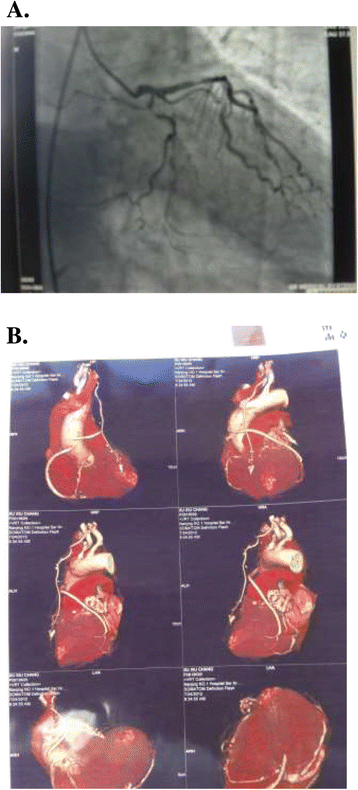

Supplement: Supplementary file 4 — Authors’ original file for figure 4 [file 13019_2014_186_MOESM4_ESM.gif]
